# Nutritional Barriers to the Adherence to the Mediterranean Diet in Non-Mediterranean Populations

**DOI:** 10.3390/foods13111750

**Published:** 2024-06-02

**Authors:** Tobili Y. Sam-Yellowe

**Affiliations:** 1 Graduate College, Canisius University, 2001 Main Street, Buffalo, NY 14208-1098, USA; t.sam-yellowe@csuohio.edu; 2 Department of Biological, Geological and Environmental Sciences, Cleveland State University, 2121 Euclid Avenue, Cleveland, OH 44115, USA

**Keywords:** Mediterranean diet, dietary pattern, food barriers, nutrition education, public health nutrition, food insecurity, racial/ethnic minorities, diet of Crete, Blue Zone diet, Native American diet

## Abstract

Adherence to the Mediterranean diet has been shown to lower the risk of developing chronic non-communicable diseases like cardiovascular and neurodegenerative diseases and cancer. Improvements in depression, participation in daily activities in older individuals, weight loss and a reduction in adverse pregnancy outcomes are associated with adherence to the Mediterranean diet. The number of studies that have evaluated barriers to adherence to the Mediterranean diet in the US and, in particular, in racial and ethnic minority populations within the US are few. Among Native American and Alaskan Native populations, studies evaluating traditional or alternative Mediterranean diet adherence for chronic non-infectious diseases is unavailable. Mediterranean diet scoring instruments used in studies in European and Mediterranean countries and among white participants in the US fail to capture the dietary patterns of racial and ethnic minority populations. In this narrative review, the food components of the traditional Mediterranean diet are discussed, adherence to the Mediterranean diet is examined in Mediterranean and non-Mediterranean countries and barriers preventing adherence to the Mediterranean diet in the US and among racial and ethnic minority populations is reviewed. Recommendations for improving nutrition education and intervention and for increasing adherence and cultural adaptions to the Mediterranean diet are provided.

## 1. Introduction

The Mediterranean diet is a food eating pattern associated with the cultures living around the Mediterranean basin. Countries such as Albania, Algeria, Bosnia, France, Greece, Israel, Italy, Morocco and Spain are part of this large region that extends into the Middle East [1,2]. Great similarities exist in the foods consumed by communities in the region. However, differences in cooking styles, herbs and spices used and influences from religion and culture contribute to differences in the diet [1,2,3]. The term “Mediterranean diet” is termed a misnomer due to variations in the foods, different cultural factors and the environment associated with the diet and the different countries around the Mediterranean region that have similar dietary patterns [4,5]. Ancel Benjamin Keys coined the name “Mediterranean Diet” upon learning about the eating patterns and food components of this diet in his studies and recognizing the association of these eating patterns with lower levels of chronic diseases associated with aging in the populations that enjoy this diet [2,5,6].

The Mediterranean diet was introduced to modern medicine through the work of Keys in his Seven Countries Study, which demonstrated the cardioprotective effects of the diet and the contributions of the diet to longevity in the communities eating the traditional Mediterranean diet [7]. The countries included in the study were the United States, Finland, the Netherlands, Italy, former Yugoslavia, Japan and Greece [7]. In the Seven Countries Study, Keys sought to understand the contributions of dietary fat to the development of cardiovascular disease. Results of his study showed that the foods of the island of Crete in Greece represented the traditional diet of Greece and that Crete had the lowest incidence of coronary heart disease, the lowest death rate and the highest longevity among the seven countries studied [7]. A study based on the French diet (which is similar to the Mediterranean diet) and the “French Paradox”, known as the Lyon Diet Heart Study [8] was conducted to investigate the relationship between diet and coronary heart disease. A 50% reduction in new acute coronary episodes as well as a reduction in new cancers and all-cause mortality was observed among the study participants. The relationship between eating patterns, in particular the association of dietary fats and the risk of chronic non-infectious diseases was established by these major studies [7,8]. Additional studies confirmed the protection observed against obesity, cardiometabolic diseases, cancers and neurodegenerative diseases [2]. The Mediterranean diet and the Lyon diet have been demonstrated to provide substantial health benefits and to prevent diseases stemming from chronic inflammation [2,7,8].

Adherence to the Mediterranean diet is decreasing in Mediterranean countries due to the increased acceptance of Western dietary patterns that promote the consumption of processed foods, pastries, red meat and sugary beverages [9,10]. The Mediterranean diet is considered a “white diet”, and the diet is thought to be marginalizing to non-white cultures and not representative since data are lacking from cross-cultural comparative research and there is a lack of diversity among research study participants in the studies that have been conducted [11]. Dietary recommendations may not take into account poor access to healthy foods in areas where food deserts and food swamps exist and where adherence to the Mediterranean diet may be low [12]. Might these controversies and challenges represent factors contributing to the low adoption and adherence to the Mediterranean diet among racial and ethnic minorities in the US? Most studies investigating the beneficial effects of the Mediterranean diet on disease prevention have been conducted in Northern Europe, Australia, Mediterranean countries and among white populations in the US. There is a dearth of information regarding studies in African, Asian and South American countries. Within the US, very few studies have been conducted among racial and ethnic minority populations [13], and a search for Mediterranean diet adherence among Native Americans and Alaskan Natives yields no results. If recommendations are being made for the adoption of the traditional or alternative Mediterranean diet, and if the diet is being prescribed for individuals, then barriers preventing adoption and adherence to the Mediterranean diet need to be identified. Otherwise, adoption of and compliance and adherence to the diet will be poor.

Nutritional recommendations that include the adoption of the Mediterranean diet are not achieving the desired goals of preventing non-communicable chronic diseases in populations most at risk for developing metabolic syndrome and other cardiometabolic conditions [14,15,16]. Barriers to the adoption of and adherence to the Mediterranean Diet in the US have not been sufficiently investigated [13,14,17], and controversies regarding the lack of cultural and ethnic diversity in the foods and study populations represented in Mediterranean diet studies create further barriers to the acceptance and adoption of the diet by racial and ethnic minority populations [11,18].

The hypothesis that food components of the Mediterranean diet are palatable, widely and easily accessible, acceptable, and affordable for individuals will be evaluated in this review paper. This narrative review will examine the barriers to the adoption of and adherence to the Mediterranean diet in the US. Barriers such as food access, food composition, food palatability, cultural foods and nutritional quality that influence the low adoption of and adherence to the diet among racial and ethnic minority populations in the US will be discussed. In particular, barriers to adoption and adherence in adults will be examined among racial and ethnic minority populations in the US. The Mediterranean diet has garnered controversy due to its origins and its non-inclusive and non-diverse research study populations, eating patterns and food components [11,13,18]. Understanding the barriers that exist will inform the nutritional counseling of clients, identify appropriate food components to include in eating plans and allow for adaptations to the diet to include nutritionally equivalent food components from other cultures in the diet. This knowledge will also help to diversify participant enrollment in research studies.

## 2. Methods

In order to identify a broad range of studies reporting on adherence to the Mediterranean diet, PubMed, Google Scholar, Cochrane Reviews and the Native Health Database were searched from the period of February 2023 to March 2024. Papers published in English, identified in the databases, were used for this narrative review. The following search terms were used: “Barriers to the Mediterranean diet”, “Non-adherence of the Mediterranean diet in the US”, barriers to adherence of the Med diet”, “food security”, “food insecurity”, “Mediterranean diet”, “Nutrients in the Mediterranean diet”, “Foods in the Mediterranean diet”, “Food insecurity and the Mediterranean diet”, “Blue Zones and the Mediterranean diet”, “Food insecurity among Native Americans”, “Native Americans and the Mediterranean diet”, “Alaska Natives and the Mediterranean diet”, “Three sisters of agriculture”, “Nicoyan foods” and “Food deserts and the Mediterranean diet”. The search terms used aimed to capture a wider range of studies that would have been missed under broader categories such as “financial barriers”, “food access barriers”, “food cost barriers” or “seasonal foods”. Due to the small number of studies reporting perceived barriers to and facilitators of the Mediterranean diet among racial and ethnic minorities in the US, papers were not eliminated. Similarly, the number of studies reporting perceived barriers and facilitators among non-Mediterranean countries outside of Europe were included because the numbers of papers were few. Also, papers were included if they discussed foods reported in questionnaires in studies investigating the Mediterranean diet and the risk of developing chronic non-infectious diseases and other conditions affecting daily living activities.

## 3. What Is the Traditional Mediterranean Diet?

**Figure 1:**
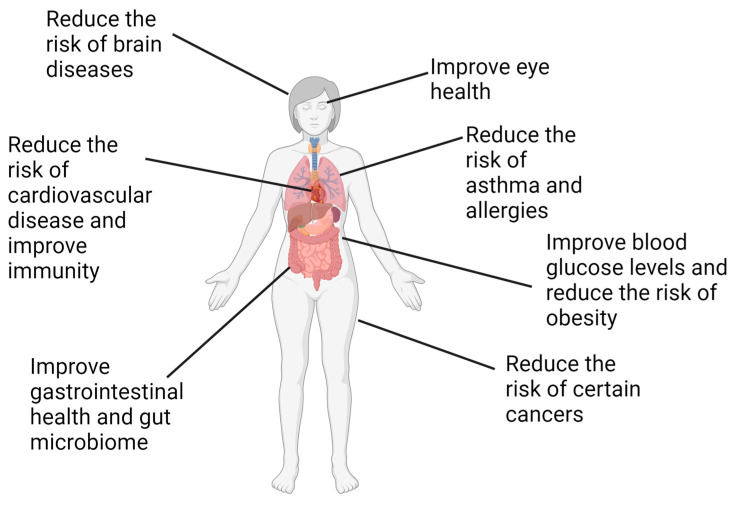
Protective effects of the Mediterranean diet (figure created with BioRender.com, accessed on 29 May 2024) [2,5,26].

What we know as the traditional Mediterranean diet originated in ancient Greece, with the connection between diet and health recognized by Hippocrates (460–370 BC). The aphorism “Let food be thy medicine and medicine thy food” [3,5] serves as the foundation for many current nutrition intervention programs. The core of the diet in the various regions of the Mediterranean basin, where olives were grown, was wheat, olive oil and wine [5,19]. Vegetables were a major contributor to the diet, and the numerous wild vegetables (known as wild greens, *Ta chorta* or *Chorta*), including purslane, used fresh or dried in a variety of dishes consumed, are recognized as an important source of omega-3 fatty acids such as alpha linolenic acid (LNA) as well as of folate, the antioxidants vitamins C and E, and glutathione [4,20,21,22]. The ubiquity of purslane and other vegetables in the Greek diet was so abundant that the Greeks were nicknamed “leaf chewers” [5]. The traditional Mediterranean diet, represented by the eating pattern of Crete, was mostly plant-based, consisting of whole grains, legumes, vegetables, fruits, nuts, moderate fish and seafood consumption, moderate dairy, small amounts of red meat and red wine. The wine was consumed with meals in small amounts [4]. Dietary fats obtained from plant and animal foods are high in omega-3 (n-3) fatty acids and low in omega-6 (n-6) fatty acids like linoleic acid (LA) [4]. Snails, consumed in the Cretan diet, are high in omega-3 fatty acids, and the poultry, mostly chicken, was free-range and fed on grass, purslane, dried figs and insects, resulting in eggs that were also high in omega-3 fatty acids [4]. The diet was balanced in the ratio of n-6 to n-3 fatty acids and had diverse phytonutrients [2,4,23,24]. This eating pattern was associated with people living in low socioeconomic conditions in Greece who ate fresh foods that used high amounts of olive oil, with little to no saturated or trans fats from red or processed meats and no commercially processed foods [6,25]. The diet provided nutrients with major health benefits and was associated with a reduction in non-infectious chronic diseases, neurodegenerative diseases and depression [2,5,26]. The Mediterranean diet has gained global recognition as an eating pattern of great value to medicine and as an important preventive measure and treatment for obesity, metabolic syndrome and related conditions such as cardiovascular disease, type 2 diabetes and neurodegenerative diseases [2,26] (Figure 1).

The United Nations Educational, Scientific and Cultural organization (UNESCO) considers the Mediterranean diet an *Intangible Cultural Heritage of Urgent Safeguarding* [2,5,27]. The United States Department of Agriculture and Health and Human Services Guidelines (2015–2020 Dietary Guidelines for Americans, 2015) (https://health.gov/sites/default/files/2019-09/2015-2020_Dietary_Guidelines.pdf, accessed on 9 May 2024) endorses the Mediterranean diet as one of the healthiest diets for preventing chronic non-infectious diseases such as metabolic syndrome, obesity, cancers, stroke and cardiovascular diseases. Do the “modern Mediterranean diets” currently promoted bear any resemblance to the traditional Mediterranean diet of Greece or Italy? Burt [11] writes that the Mediterranean diet has been “white-washed” to omit foods that are not palatable or familiar to white consumers, while downplaying the importance of the omitted cultural foods to the populations that consume them, such as beans, pulses, teff, and cassava (yucca), which are also consumed in the Mediterranean region. This type of omission might further discourage individuals from adopting the Mediterranean diet, particularly if those omitted foods are part of their cultural dietary patterns. Moreover, omission of foods such as red meat and discouraging the daily consumption of foods such as eggs, poultry and fish may not be an accurate representation of the eating patterns in Italy and Greece during the 1960s [3,11].

The Mediterranean diet entered mainstream usage and is now synonymous with good health and longevity since Ancel Keys first introduced the term in 1975 [1,28]. Different regions of the Mediterranean consume similar food components produced, prepared and cooked in similar or different ways, depending on cultural and religious influences, leading to the argument that the Mediterranean diet is not unique to one country, unlike the association made with Greece, specifically with Crete [1]. Confusion exists regarding what constitutes the Mediterranean diet, leading to inconsistencies in the types of foods that are self-reported in studies. Is eating a single food item from the Mediterranean diet, such as olive oil, sufficient to claim adherence to the diet? Many of the studies evaluating the Mediterranean diet in association with various health conditions do not assess food components of the traditional Mediterranean diet since there are variations of the diet in the Mediterranean region [5,29]. For example, food components such as vegetables, when described in studies on self-reported questionnaires as a major component of the Mediterranean diet, refer to cultivated vegetables. The traditional Mediterranean diets, as described for Greece and Italy, contain numerous wild vegetables consumed daily in different meal preparations that have been known from ancient times, and they continue to be consumed in the present time in both countries [20,21]. These ubiquitous vegetables constitute a major component of daily meals, providing health benefits that reduce the risk of disease. Many of the vegetables have no English names and are often local to communities in Greece and Italy and to other Mediterranean countries [20,21]. In addition to the wild vegetables, the use of herbs and spices such as basil, bay leaf, fennel, cloves, thyme, rosemary, cumin, turmeric, ginger, mint, parsley, garlic, sage, oregano and cinnamon [30,31] adds to the nutrient density of the Mediterranean diet. However, the region that is most associated with the Mediterranean diet is the island of Crete. Therefore, an examination of the traditional diet of Crete from the 1950s and 1960s is warranted to understand what is meant by the “Traditional Cretan Diet”, which is what the traditional Mediterranean diet is based on and for which the health benefits and food taste or palatability have been described in association with the work of Ancel Keys [32]. The role that wild vegetables play in the traditional diet is critical and has been described as the “hidden MD” [22,33]. Before examining why there would be barriers to the Mediterranean diet and what foods are considered unfamiliar or unpalatable, it will be helpful to have a knowledge of how different the foods were in the 1960s compared to food components that are available in 21st century grocery stores and farmers’ markets in the US. The emphasis on fresh and unprocessed foods that are prepared, cooked using moist cooking methods and eaten right after cooking is part of the traditional Mediterranean dietary pattern [3]. Furthermore, meals were eaten with family or friends rather than alone in front of electronic devices or “on-the-go” [3]. Specific descriptions of “homemade minestrone”, vegetables (“leaves”) eaten with olive oil, beans, pasta with tomato sauce and cheese, freshly baked bread consumed without “spreads”, moderate amounts of meat or fish, fresh fruit and red wine known as Dago red constitute the eating patterns of the traditional Mediterranean diet as described by Noah and Truswell [1] and Russo et al. [34].

In the traditional Mediterranean diet, there are foods native to the Mediterranean region and some foods that have become associated with the Mediterranean diet that have come from other cultures, such as from Africa (e.g., okra, watermelons, artichokes), Asia (e.g., buckwheat, chickpeas, lentils, garlic, turmeric) and the Americas (e.g., eggplant, tomatoes, corn, potatoes, zucchini, coffee) [2,3]. Whole grains, olive oil and wine are considered major traditional components of the Mediterranean diet [5,19]. Furthermore, foraged (wild) vegetables along with fruits, legumes and moderate fish consumption are part of the traditional diet. Just as there are many versions of the Mediterranean diet, there are variations in what has been reported in the literature as the traditional diet of Crete. Recipes in cookbooks reflecting the foods consumed in ancient Greece up to the 1950s and 1960s show the use of cabbage, spinach, Swiss chard, okra, eggplant and potatoes [19] (Table 1). The vegetables are described as “cooked swimming in oil”, the oil being olive oil [19].

These food examples are provided in this review to highlight what I consider the “awareness factor”. If individuals are aware of what the traditional Mediterranean diet consists of, if necessary, rational substitutions can be made to incorporate other cultural foods and cooking styles. Furthermore, with nutritional education that includes meal preparation techniques, individuals may accept the Mediterranean foods as they are, without a need for changes.

Mediterranean diet food pyramids have changed several times, along with the recommendations for servings for fruits and vegetables, legumes, nuts, fish, seafood and poultry, compounding the confusion that individuals face when trying to understand and eat the Mediterranean diet [35,36]. Many foods considered traditional to the Mediterranean diet are also found in other cultural cuisines, such as tomatoes, chili peppers and wheat flour. However, foods such as olives and olive oil are the hallmarks of the traditional Mediterranean diet [30,37,38]. Kafatos et al. [39] described the traditional diet of 1960s Crete and compared it to a typical diet of adolescents and a fasting diet of the Eastern Orthodox Church. Nutrient values were also evaluated. Breakfast meals were simple but nutrient-dense and included olives, and a typical lunch and dinner included legumes (chickpeas, broad beans, lentils), a variety of vegetables and fruits, fish and salads. Mid-morning and afternoon snacks included fruits and nuts, homemade cheese, pie, honey and coffee [39].

Components of a Greek diet reported by Trichopoulou et al. [32] for a weekly menu showed similar food items as reported by Kafatos et al. [39]. However, notable differences include the inclusion of herbal tea, sugar and bread in the breakfast meals 6 out of 7 days, daily sugar intake with the afternoon snack along with Greek coffee and bread every day for lunch and dinner. Feta, black and green olives, legumes and red wine were included with meals. Fish, chicken, roast lamb and fruits (apple, pear and orange) were also part of meals [32]. Dinner meals were similar to lunch [32]. Except for snails, which are not typically found in American meals, the other listed food items may be found in local farmers’ markets or grocery stores. Radd-Vagenas et al. [3] describe meal patterns and food components similar to those described by Kafatos et al. [39] and Trichopoulou et al. [32], demonstrating important similarities in the foods consumed in the 1950s and 1960s in Crete. Very little red meat was consumed [32,39]. How often would a regular consumer purchase these food items from a US grocery store or farmers’ market for daily food preparation and consumption? Nutrition knowledge may provide the answers.

## 4. Blue Zone Diets

Due to the similarities in the influence of the Blue Zone diets and the Mediterranean diet on health and longevity, a comparison of the Blue Zone diet with the Mediterranean diet is discussed in this review. Blue Zone dietary patterns are often confused with the Mediterranean diet. However, the importance of the Mediterranean diet does not rely on a single food component but on the synergy of the different food components in meals and the social and cultural environment surrounding the acquisition of the food, the preparation and cooking of the food and the enjoyment of the food in the company of a “community” of family and friends. A missing component in the benefits of the Mediterranean diet and perceived barriers that may exist toward acceptance of the diet may include the need for “community”, which is challenging to replicate in the very mobile society of the US, where most individuals eat on-the-go and frequently eat alone in front of the television or other screen device. Buettner and Skemp [40] described the factors contributing to longevity and the reduced incidence of neurodegenerative and non-infectious chronic diseases in five regions of the globe that are designated Blue Zones. These regions include Loma Linda, California; Nicoya, Costa Rica; Sardinia, Italy; Ikaria, Greece and Okinawa, Japan. In these zones, people live to the age of 100 ten times more often than in the US [40].

The diet of Ikaria closely resembles the Mediterranean diet of Crete in the 1960s as described by Ancel Keys (1980) [7]. Many of the benefits of the Mediterranean diet, in particular the types of vegetables, fruits, and olive oil that make up the major food sources, are found in the Ikarian diet, resulting in similar health benefits to the population [40,41]. Along with the foods consumed in the Blue Zones, nine key characteristics known as “Power 9” have been defined as characteristic to the way of life in the Blue Zones and include strong community support in daily living [40]. These characteristics include natural movement, having a purposeful life, the ability to relieve stress naturally (“downshifting”), eating to 80% satiation, eating a mostly plant-based diet rich in legumes, drinking wine in moderation with food or with friends, belonging to a faith-based community, placing family first and belonging to a committed and supportive social circle that practices healthy behaviors [40]. These characteristics are similar to the lifestyle in Crete. Although there are similarities, there are particular food items that are unique to each Blue Zone and that are different from the foods found in Crete (Table 2).

Calorie restriction is built into the dietary practices in each of the Blue Zones, similar to the small to moderate portion sizes of meals described in the traditional Mediterranean diet [42,43]. The reduced oxidative load, combined with foods rich in antioxidants, is thought to contribute to the longevity in each of the Blue Zones. In each of the zones, meat consumption is moderate and in small amounts of three to five ounces and occurs about five times a month. Whole-grain cereals, legumes, vegetables and a variety of fruits make up the core diet [40]. The diet of the Blue Zones and the Mediterranean diet are rich in polyphenols and omega-3 fatty acids [40]. The traditional Sardinian diet consists of whole-grain bread, beans, garden vegetables, fruits and potatoes, with meat consumption on Sundays and on special occasions. Moderate drinking of Cannonau wine, which is rich in flavonoids, is thought to help alleviate stress [44]. The Ogliastra subregion of the island of Sardinia is reported to have the longest-lived men [44]. In Ikaria, the diet is a variation of the traditional Mediterranean diet, with fruits and vegetables, whole grains, beans, potatoes and olive oil making up a large amount of the food consumed. In addition, the consumption of wild greens contributes to the overall benefits of the diet [41]. Mid-afternoon breaks and naps contribute to reduced stress, a pattern also observed in Crete, and is associated with reduced blood pressure and coronary mortality [3]. In a study of the oldest old people (71 participants > 90 years of age) in Ikaria, 62.7% adhered to the Mediterranean diet and had low rates of diabetes and alcohol consumption along with very low levels of cardiovascular risk factors [45]. The diet of the Nicoyans consists mostly of unprocessed foods and includes legumes, cereals, antioxidant-rich tropical fruit, milk, coffee, potatoes, corn, rice, beef, fish, light cheese, and sweets and sodas in moderation [44,46]. The water in Nicoya is rich in calcium and magnesium [44], and the availability and contributions of these two important micronutrients are important characteristics for the overall health and longevity of the Nicoyans. Fish is consumed, with males consuming less than females, and dairy is consumed less in males than in females. Faith, family and a purposeful life, *plan de vida*, are part of the lifestyle on the Nicoyan peninsula [44,46]. Longevity related to a diet resembling the Blue Zone and Mediterranean diets, lifestyle and environment was observed in Bama, China [47] and in the Amami Islands, Japan [48]. The high mineral content of the soil and hard-water characteristics of the Amami Islands may also provide important cardioprotective benefits to residents [44,47,48]. In the US, the Adventists have active daily lives and consume a vegetarian diet that ranges from vegan to lacto-vegetarian, rich in green leafy vegetables, nuts and legumes [49,50]. They recognize the Sabbath, and “downshift” for twenty-four hours every week [40]. The Okinawan diet is low-calorie with reduced fatty acid consumption [51,52]. The diet is rich in seaweed (sea vegetables), Okinawan tofu, bitter melon, lots of vegetables, soy products, sweet potatoes, turmeric, seafood such as fish, and pork [53,54,55]. The longest-lived women are found in Okinawa. The practice of Moai, which places individuals in secure social networks formed from as early as the age of 5, the 80% rule of *Hara Hachi Bu* repeated at mealtimes to prevent overeating and a strong sense of purpose contribute to the longevity and the reduced levels of neurodegenerative disease and other diseases of inflammation [53,54,55]. A high number of centenarians are found in the Amami Islands in Japan, which are characterized by the availability of hard water rich in calcium and magnesium, considered an important factor contributing to longevity among residents [48]. All five Blue Zones share the same nutrients found in the Mediterranean diet that benefit health and that contribute to longevity, and all the zones have in common foods, calorie restriction associated with small to moderate food portions and cultural practices that are unfamiliar to populations outside the zones; in particular, populations in the US may associate or interchange the Mediterranean diet with Blue Zone diets.

The endorsement of the Mediterranean diet by the United States Department of Agriculture and Health and the Human Services Guidelines (2015–2020 Dietary Guidelines for Americans, 2015) has led to the development of nutrition intervention programs requiring individuals to change dietary patterns and habits to adopt this mostly plant-based eating pattern. For individuals used to processed and pre-packaged foods and being unfamiliar with the components of the Mediterranean diet, such a change appears daunting. The benefits of the diet are to be found in the nutrient-dense quality of the foods, which are consumed raw in the case of vegetables used in salads. When the vegetables are cooked, olive oil is used liberally as it is also in the salads. The major nutrients abundant in the fruits and vegetables are polyphenols, which have important roles in metabolism, cell proliferation, weight management and have antioxidant, anti-inflammatory and anti-proliferative properties [56]. The evidence-based health benefits of the phenolic nutrients, reported for the prevention of chronic non-infectious diseases such as metabolic syndrome, obesity, cancers, stroke and cardiovascular diseases, are well documented [23,56,57,58]. More than 8000 phenolic phytonutrients are produced only by plants [59] and include the two categories of flavonoids and non-flavonoids based on the chemical structure of the compounds. Among the flavonoids are the flavanols, flavonols, flavanones, isoflavones, flavones and anthocyanins [35,59]. The lignans, tannins, stilbenes and coumarin make up the non-flavonoids [59]. The functions of these phytonutrient compounds include scavenging for free radicals such as reactive oxygen species, thereby protecting nucleic acids, lipids and proteins in cells, reducing inflammation, regulating the redox state of cells and contributing to the epigenetic modulation of gene expression in cells [59]. The protective role of the Mediterranean diet, which is rich in antioxidants and anti-inflammatory nutrients, mirrors the same beneficial effects that have been shown to protect against cardiovascular disease, metabolic syndrome, cancers and other non-communicable chronic diseases [58,59].

## 5. Adherence to the Mediterranean Diet in Mediterranean Countries

The Mediterranean diet has continued to gain widespread acknowledgement as a healthy diet that reduces the risk of many chronic non-communicable diseases. However, adherence to the diet in the Mediterranean region has been declining, even as levels of obesity have increased [9,10,60,61]. A review of Mediterranean diet adherence in Mediterranean countries identified fifty studies, in which thirty-five reported low to moderate adherence, and highlighted the small number of studies that have been conducted among the African Mediterranean countries of Egypt, Libya, Tunisia, Algeria and Morocco [9]. In the fifty years since Ancel Keys reported his study, individuals in the Mediterranean region have incorporated more Western-style food components into their diet, and obesity levels have increased steadily [60]. The increase in saturated fats obtained from processed and packaged foods, red meat consumption, packaged pastries, sugary beverages and a higher salt intake have reversed many of the benefits of the traditional Mediterranean dietary pattern [34]. Fruit and vegetable consumption have reduced, including the use of olive oil as the major lipid source for monounsaturated fatty acids. Along with a reduction in fiber and polyphenols from a low intake of fruits, vegetables and legumes and a more sedentary lifestyle, many of the gains associated with the traditional Mediterranean diet are being eroded [10,34]. Initial shifts from the traditional Mediterranean diet have been reported in the Mediterranean countries in Europe and in the Southern Mediterranean region [10]. Changes in food preferences, food costs and industrialized food production have been implicated in the decline in Mediterranean diet adherence [34]. A study of Spanish older adults showed only a 23.7% adherence to the Mediterranean diet, with an increased intake of fast foods, sugary beverages and commercial pastries and a reduced consumption of legumes, fruits, vegetables, cereals and fish [62]. In the study, the differences between the levels of education of individuals were not significant [62]. Barriers to adherence to the Mediterranean diet in the Spanish population included sensory qualities such as food taste, lengthy food preparation times (which have led to a reduction in the amount of legumes consumed) and the food cost [62]. Adherence to the Mediterranean diet in the Mediterranean countries was investigated for sex differences in adherence, but the results remain inconclusive [9]. A study of Greek students found low adherence to the Mediterranean diet, with students studying dietetics and female students having a higher Mediterranean diet adherence [63]. Adherence was associated with increased food costs, and the study identified food insecurity among the Greek students, which also influenced the low adherence and the choosing of unhealthy foods [63]. Low adherence was reported in a study of rural Lebanese, where a shift in dietary patterns was observed, with an increased intake of refined cereals, liquid sweets, fats and oils and a reduced consumption of olive oil, whole cereals, legumes, fish, red meat and poultry [61]. The association of the Mediterranean diet with academic performance in public and private school students showed significant improvements in academic achievement as a result of better health and overall improved cognition [64]. A study evaluating Mediterranean diet adherence in a Jewish population of men and women in Israel found a low consumption of olive oil due to the cost and a similar intake of fruits and vegetables when compared to other Mediterranean countries [65]. Low consumption of Mediterranean diet food components was associated with cardiovascular disease [65]. Investigations of adherence to the Mediterranean diet in Israeli adolescents of middle-school age identified low adherence in the Mabat Youth Survey 1 [66] that was associated with the education level of mothers and the inadequate reading of food labels. A follow-up study found increased adherence and improved Mediterranean Diet Quality Index (KIDMED) scores [66], suggesting that improvements can be made with a better understanding of food labels and food selection [67]. Declining Mediterranean diet adherence was also reported in Croatia, where a low intake of fruits and vegetables, olive oil, red wine, fish and sea food was also observed [68]. Adoption of Western-style dietary patterns and the high cost of Mediterranean diet food components influenced adherence [68].

## 6. Barriers to Adoption of and Adherence to the Mediterranean Diet

Adoption of and adherence to the Mediterranean diet has been studied in countries around the Mediterranean region, in other parts of Europe and in countries outside Europe, including the US [10,69,70]. Various factors serve as facilitators and barriers to the adoption of dietary changes in different communities. Tsofliou et al. [15] describe eight categories of barriers identified among the studies reviewed. Among the categories with the most barriers are cognitive, motivational, financial, sensory and hedonic factors [15]. Socioeconomics contribute to the types of food purchases made by individuals. A higher income, a high level of education, access to healthy foods and knowledge of dietary components such as those in the Mediterranean diet are associated with high adherence [71]. Living in areas with food deserts, a low income, a low level of education and unfamiliarity with the components of the Mediterranean diet are associated with low adoption and adherence [71]. Unfamiliarity with cooking styles, food flavors and food culture can also contribute to hesitancy in adopting and adhering to the diet. Due to the abundant evidence for the health benefits of the Mediterranean diet, reasons for non-adoption of and non-adherence to the Mediterranean diet have been studied by investigators. Adherence to the Mediterranean diet was investigated with respect to the cost of food components in Extremadura, Spain, a region with the lowest per capita income in the country. This region is also reported to have the highest rates of morbidity and mortality from cardiovascular disease [71]. Increased adherence was observed in areas of higher income, with increased expenditure for the types of foods consumed in the Mediterranean diet compared with individuals consuming a “Western food pattern” that was seen to be of lower cost [71]. Similar association of higher dietary cost with high adherence to the Mediterranean diet was reported in the Fenland study conducted in the UK [72]. High income and education were associated with high adherence [72]. Healthy food purchases, including of vegetables, legumes, fish, nuts, cereals and olive oil, were associated with higher expenditure, while those foods considered unhealthy, such as red and processed meat, potatoes and sweets, were low-cost and affordable [72].

In spite of the global recognition of the health benefits and of its contribution to disease protection, adherence to the Mediterranean diet has been declining in Mediterranean countries, such as in Greece and Italy, and in non-Mediterranean European countries since the 1960s [10,73]. Adherence to the Mediterranean diet in the US is also reported to be low, despite studies showing its protection against cardiovascular disease, cancers, metabolic syndrome, obesity and excessive weight gain during pregnancy [16,17]. Various reasons have been proposed as perceived or true barriers to the adoption of and adherence to the diet among different non-Mediterranean populations, including populations in the US [16,17]. Reported barriers include a lack of familiarity with food components of the diet, food taste, cost of the diet components, culture and eating traditions, food availability, knowledge of the Mediterranean diet, lack of time to prepare and cook foods for the diet, sensory and hedonistic barriers and the seasonality of food components [15,17]. In addition, nutrition knowledge and higher education are reported to increase adherence to the Mediterranean diet, with low rates of obesity [74]. Due to conflicting ideas regarding what constitutes the Mediterranean diet and what food components should be consumed in the diet, individuals may be adding foods to their diet, albeit at low amounts, without realizing that they are consuming food components of the Mediterranean diet [75]. This can lead to challenges in assessing barriers to the adoption of and adherence to the Mediterranean diet among different populations in the US.

## 7. The Mediterranean Diet in Non-Mediterranean Countries

Studies examining the adoption of and adherence to the Mediterranean diet have mostly been conducted in countries in the Mediterranean region, Europe, Australia and white populations within the US [11,13,15,16]. A comparison of Mediterranean diet food components to foods of similar nutritional composition in other countries shows that many foods exist in countries on all continents that can be adapted to a modified Mediterranean diet but are not due to barriers in food taste and availability perceived by individuals outside the Mediterranean region [69]. Very few studies examining knowledge of, and adherence to, the Mediterranean diet have been conducted in African countries, central and South America and among Caribbean countries. Efforts to adapt the Mediterranean diet with other cultural cuisines is seen in the Indo-Mediterranean diet, using aspects of the Mediterranean diet but including foods and spices found in Indian cooking such as millets, brown rice, turmeric, cinnamon and cumin [76]. Antioxidant levels are high in the Indo-Mediterranean diet. However, a major difference between the Indo-Mediterranean diet and the Mediterranean diet is the lack of animal foods in the Indo-Mediterranean diet. Fish is consumed, but saturated and total fats, salt and sugar are low while omega-3 fatty acids and flavonoids levels are increased [76]. Many studies investigating adherence to the Mediterranean diet do not use foods found in the traditional Mediterranean diet [29,69]. Foods consumed and dietary patterns adhered to by racial and ethnic minority populations in the US do not contain some of the types of foods included in the traditional Mediterranean diet [13]. In the reported studies regarding adherence to the Mediterranean diet, scoring instruments used to determine nutrient uptake reflect the foods found in the traditional Mediterranean diet as originally designed by Trichoupolou et al. [77], yet these foods are not typically consumed by study participants. These foods may be consumed by white participants but not by individuals in racial and ethnic minority populations, and if these instruments are used in studies, the diet scores obtained will not accurately represent a nutrient uptake that reflects the traditional Mediterranean diet [11]. The diet scoring instruments are also used in studies that enroll participants from Europe, Australia and the countries of the Mediterranean regions, further highlighting the potential skewing of data resulting from their use in the few studies that have enrolled racial and ethnic minority participants in studies [11]. Cultural differences in food components and dietary habits play a large role in the non-adoption of and non-adherence to the Mediterranean diet [15,78]. The high cost of foods consumed in the traditional Mediterranean diet are seen as a barrier to adoption and adherence for individuals with low median household incomes living in low socioeconomic communities, according to the Fenland study of participants from the UK and in a Northern European population [72,79]. These barriers exist despite the health benefits of the diet. In the Northern European study, additional food-related barriers were food availability and expense, eating habits, taste, living in a cold climate and a lack of knowledge and cooking skills [80]. Red meat consumption in the Mediterranean diet is low [19,32,39] and is seen as a major barrier for individuals who regularly consume red meat. Food components such as olive oil, fish, fruits, vegetables and nuts are considered expensive, and although legumes may be affordable, the cooking methods and food tastes may not be palatable [15]. Whether these differences extend to actual metabolic or physiologic differences in the effects that the foods have on health is an important area for further investigation. Food types, cultural differences and unfamiliarity with foods in the Mediterranean diet have created barriers to adoption and adherence. In many of the studies conducted to evaluate adherence to the diet in association with non-communicable chronic diseases, in particular when intervention studies were performed, modifications to the diet were made [13], and in responses provided for questionnaires, self-reported entries show that some food components are not found in the traditional Mediterranean diet [13,81,82,83,84,85]. Perceived barriers reported among study participants in non-Mediterranean countries also differ in the factors leading to non-adherence. Food costs were considered a barrier to adhering to the Mediterranean diet in the UK Fenland study [72]. Vegetables, legumes, fruits, nuts, fish, cereals and olive oils were associated with high costs compared with the foods typically considered unhealthy, such as red and processed meats, potatoes and sweets [72].

The health benefits of the Mediterranean diet have been replicated in non-Mediterranean countries [72,73,74,86]. However, knowledge, availability and acceptance of the traditional food components of the Mediterranean diet remains a major challenge. Factors presenting major barriers to adherence to the Mediterranean diet include knowledge, convenience, sensory appeal and health [15,72,86]. In an Australian study, high adherence to the Mediterranean diet was associated with low cardiovascular disease risk factors [86]. Among Australian older adults, barriers to adhering to the diet include the lack of variety in the diet, low consumption of red meat and food tastes, in particular toward natural Greek yogurt, which is a major food component of the Mediterranean diet [35]. The complexity of the Mediterranean eating pattern, individual food preferences and perceived cost were major barriers to adhering to the Mediterranean diet in another Australian study [87]. Older Greek-born Australians adhered to the Mediterranean diet with increasing age, resulting in protective effects against cardiovascular disease risk factors [31]. The reduction in salt intake and increased use of aromatic herbs and vegetables, spices and the predominance of olive oil in the diet influenced many of the observed health benefits and palatability of the diet [88]. The anti-inflammatory and antioxidant benefits of the food components in the Mediterranean diet positively impact health and reduce the risk of non-infectious chronic diseases [31,88].

Another Australian study enrolled 606 participants and used the Theory of Planned Behavior Framework to assess the perceived barriers to and facilitators of adherence to the Mediterranean diet [86]. Participants included Australian males and females aged 18 years and older. Respondents to the 14-item Mediterranean Diet Adherence Screener (MEDAS) reported middle to high income and identified depression and arthritis as the most prevalent health conditions [86]. A large number of participants (96.0%) reported health benefits, diet quality, appeal, lifestyle, affordability and the environment. However, 61.1% of participants also identified potential disadvantages in the Mediterranean diet, similar to the disadvantages reported in other studies [13,15]. Among the disadvantages noted were food literacy, the need for cooking skills, healthfulness, convenience, food taste and culture as barriers. Food purchases were thought to be expensive, with 25.1% reporting the high cost of fruits, vegetables and fresh sea food [86]. Participants reported food taste, oily foods and lack of variety as major barriers to the Mediterranean diet. Among participants, 20.3% perceived barriers in lack of food access as a major challenge to Mediterranean diet adherence [86]. Participants also identified potential reactions to food allergens and gastrointestinal conditions that could be worsened by food items such as wheat contained in the diet. The increased amounts of carbohydrates, fats and oils consumed from olive oil, nuts and animal products were seen as barriers. Only 2.6% responded that the diet was affordable, and among participants, 18.2% with higher resources reported being able to adhere to the diet [86]. Examples of barriers identified were perceived behavioral control, perceived health benefits (76.5%), improved diet quality (38.5%) and dietary adherence (39.7%) [86]. Among several studies conducted in the UK, a study of middle-aged adults reported that purchasing, organizing and preparing foods for the Mediterranean diet were major barriers toward adopting and adhering to the Mediterranean diet [17].

A cross-sectional study that enrolled Turkish medical students to evaluate knowledge of healthy eating patterns and adherence to the Mediterranean diet found low adherence to the diet among students living in dormitories [89]. The KIDMED index was used in the study, and 19% of first-year medical students and 31.3% of third-year medical students had low KIDMED scores. Increased smoking and low physical activity were associated with low Mediterranean diet adherence and less focus on healthy eating [89].

In a South African study of pregnant women aged 18 to 44 years of age, using a cross-sectional study design, participants formed a secondary study from a larger Nutritional Status of Expectant Mothers and their newborn Infants (NuEM). Mediterranean diet adherence was evaluated using the MeDAS 14-item questionnaire [90]. Adherence to the Mediterranean diet was low in many of the scored items on the questionnaire, including the low use of olive oil and low consumption of fruits and vegetables, nuts, legumes, fish, shellfish and sofrito [90]. A higher level of education and higher income were associated with slightly improved adherence. Shifts in food convenience and grocery shopping habits as well as unfamiliarity with the Mediterranean diet also influenced eating patterns, which have become more Westernized [90]. Refined grains, sugary beverages and sunflower oil are major components of the South African diet [90]. In a cross-sectional study that enrolled black diabetic participants in Kinshasa, Democratic Republic of Congo, association of the Mediterranean diet with the regular intake of vegetables was evaluated in diabetic patients with blindness, cataracts and glaucoma [91]. The Mediterranean-style dietary score (MSDPS) adapted for use in Africa was used by participants to self-report Mediterranean-style food intake. Foods in the Mediterranean diet and African fruits and vegetables (okra, plantains, cassava leaves and red beans) was associated with low risk of blindness, cataracts and glaucoma in study participants [91]. The food components of the Mediterranean diet consumed by participants were not identified, and barriers to the diet adherence were not discussed [91]. The KIDMED scoring system was used to evaluate adherence to the Mediterranean diet in a study to determine the prevalence and risk factors for hypertension in a Nigerian study [92]. Scores obtained indicating poor adherence and average to good adherence had no significant association with hypertension [92]. Consumption of foods such as oil, fish fruits, vegetables, cereals, nuts, pulses, pasta or rice, dairy products and yogurt were associated with adherence, while not eating breakfast or consuming fast foods and baked goods indicated poor adherence to the Mediterranean diet [92]. The nature of the particular foods and oil reflecting the Mediterranean diet was not specified and reflects observations from the few studies that have included African participants on the African continent.

There is also a paucity of studies in South America evaluating adherence to the Mediterranean diet. The Mediterranean diet has been evaluated in Chile to determine its associations with markers for chronic disease, disease risk factors as well as metabolic syndrome. The data showed that Mediterranean diet components in an intervention study improved abdominal obesity and arterial hypertension and lowered HDL cholesterol [93]. Specific food components of the traditional Mediterranean diet were not specified. Low adherence to the Mediterranean diet was associated with an increased risk for chronic disease in the Chilean study [94], with studies aimed at increasing plant foods for improved health [95]. Similar studies have been performed in other South American countries evaluating diabetes in Brazil using adapted measurement instruments [96,97], sleep duration interference with dietary patterns in Costa Rica [98] and muscle fitness in college students in Columbia [99].

Among countries in the Middle East, a study conducted in Iran evaluated adherence to the Mediterranean diet in association with the risk of kidney disease and found that in participants with high adherence to the Mediterranean diet, there was a 50% lower risk of chronic kidney disease [100] and a reduced risk of type 2 diabetes [101]. Increased intake of legumes, fish, seafood and nuts and an increased MUFA:SFA ratio was associated with a lower risk of type 2 diabetes [101]. In a separate study using the same cohort of the Tehran Lipid and Glucose study, a nested case–control study design within the larger cohort showed that Mediterranean diet adherence was not associated with type 2 diabetes [102]. Discrepant results like those obtained in the Iranian studies are strong indicators for the need for more population-based and nutrition intervention studies with larger study participants in countries outside the Mediterranean region. The risk of obesity and adherence to the Mediterranean diet was investigated in a cross-sectional study with participants from three Gulf countries (Saudi Arabia, Oman and Kuwait) [103]. Low adherence to the Mediterranean diet was observed in the three countries. Intakes of olive oil, fruits and vegetables were low. Higher adherence to the Mediterranean diet resulted in a low BMI and hip circumference (HC) [103]. A study conducted in Saudi Arabia to determine the association of the Mediterranean diet with immune status showed that the consumption of fruits, vegetables, nuts and fish was low compared with the increased intake of sugary beverages and processed foods [104]. Barriers to Mediterranean diet adherence include unawareness of the Mediterranean diet and its health benefits and sociocultural factors such as the presence of fish and alcohol as components of the diet that are not frequently consumed in Saudi Arabia [15,104]. However, high adherence to the Mediterranean diet was found to be associated with a favorable immune status [104]. Recognizing the need for a more accurate food questionnaire reflecting food intake in Saudi Arabia, Aljehani et al. [105] reported on a Mediterranean Diet Scale in Arabic that aims to provide more consistent and accurate participant information.

Among studies conducted in the Asian countries of South Korea, China and Japan, modifications to the Mediterranean diet food components were reported [106,107,108]. Using a modified Mediterranean diet score [77], adherence to the Mediterranean diet and association with metabolic syndrome was evaluated among Korean adults in the Korean National Health and Nutrition Examination Survey (KNHANES) 2012–2015. The Mediterranean diet score was modified to accommodate the dietary eating patterns of Koreans. For example, potatoes were not included in the vegetable category, sea mustard/laver and kelp were included in the vegetable group and fish and nuts were placed in one category [106]. The foods comprising a variety of vegetables, legumes, tofu, multigrain rice, red and processed meats, poultry, fish, dairy and alcohol were categorized into groups that reflected Korean foods and eating patterns. Beverage consumption was also modified [106]. High adherence to the modified Mediterranean diet was associated with a lower prevalence of metabolic syndrome, abdominal obesity and hypertriglyceridemia [106]. The 14-point Mediterranean diet score, translated into Chinese, was used to test the association of prediabetes with adherence to the Mediterranean diet among Taiwanese adults [107]. High adherence to the Mediterranean diet was associated with low fasting glucose and A1c levels and a lower risk of prediabetes [107]. The intake of foods typically found in Western diets such as butter, cream, margarine, sweet beverages, carbonated drinks and red and processed meats was low (less than one serving a day and less than one glass a day). Wine and olive oil were considered expensive items [107]. Adherence to Japanese dietary eating patterns and the Mediterranean diet were studied for their association with muscle weakness in middle-aged and older adults in the Japanese Survey on Aging (JSTAR) [108]. The 12-component revised Japanese diet index (rJDI12) was used to evaluate the Japanese dietary pattern using the alternate Mediterranean diet (aMED). Food components for each pattern reflected the foods typically associated with each pattern, with more fish and seafood, pickled vegetables, legumes (soybean), green tea and red and processed meats in the Japanese dietary pattern compared with the Mediterranean pattern. While there was a small similarity in the benefits of both dietary patterns among the Japanese participants, adherence to the Japanese dietary pattern was associated with the maintenance of muscle strength [108].

## 8. Adherence to the Mediterranean Diet in the US

An examination of factors serving as barriers to or facilitators of adherence to the Mediterranean diet require an understanding of the types of food and beverage intake reported by study participants in the various studies conducted regarding the benefits of the Mediterranean diet. While most of the studies were not designed to investigate factors determining adherence to the Mediterranean diet, participant responses provided information about food preferences and challenges associated with adherence to the Mediterranean diet. The responses also provide important insights regarding how the Mediterranean diet can be used in nutrition education or counseling [2,13,15,16].

A randomized controlled intervention study that enrolled mostly highly educated women in Michigan reported high Mediterranean diet adherence scores [109]. The intervention arm of the study evaluated the increase in fruit, vegetable and monounsaturated fatty acid intake in a modified Mediterranean diet using a self-selected food-exchange list, where foods available in the US of similar nutrient profiles were consumed. The non-intervention arm received some information aimed at correcting nutritional deficiencies without any counseling [109]. Although improvements in plasma carotenoid and monounsaturated fatty acid levels were observed, no changes in diabetes and cardiovascular disease (CVD) risk factors were obtained [109]. No barriers to adherence were discussed in the study. When diverse populations are examined among racial and ethnic populations, differences in adoption of and adherence to the Mediterranean diet are observed, with important health implications such as the conditions reported in the Stroke Belt and Buckle of the US [16,110].

Jaacks et al. [111] evaluated the protective effects of the Mediterranean diet on cardiovascular disease risk factors and whether supplementing a regular American diet with key food components of the Mediterranean diet would provide protective effects against CVD risk factors. The pilot randomized controlled trial conducted in Atlanta, Georgia, stratified participants into three arms and consisted of a Mediterranean diet, a typical American diet with fish oil, walnuts and daily grape juice or a typical American diet alone for eight weeks [111]. Decreases in total cholesterol and LDL cholesterol levels and in body weight were observed in the Mediterranean diet arm along with a reduced risk of CVD, and triglycerides were reduced in the supplement arm along with adiponectin reduction when compared with the American diet arm [111]. While the supplemented diet showed some improvements, CVD risk factors and weight loss were not significantly affected. During the study, prepared Mediterranean meals were provided to the participants in the Mediterranean diet intervention arm along with counseling. However, upon follow up eight weeks after the study, adherence to the diet had decreased [111]. Reasons for the decreased adherence, including any barriers that influenced adherence, were not discussed and constitutes a lost opportunity to gain valuable insights regarding factors that might contribute to non-adherence to the Mediterranean diet. Presumably, the convenience of having Mediterranean-style meals available during the study facilitated adherence [111].

Couto et al. [112] investigated Mediterranean diet adherence in a Portuguese immigrant population living in the US in California, in the cities of Turlock and Livermore. They reported differences in the level of Mediterranean diet adherence within the two communities [112]. Specific characteristics of the two communities are highlighted in this review to draw attention to common factors that influence how individuals perceive diets. These factors were captured in both populations and fall under the categories described by Tsofliou et al. [15,112]. Adherence to the Mediterranean diet, perceived knowledge and barriers were evaluated in the study. The 14-question Mediterranean adherence Diet Adherence Screener (MEDAS) was used by a convenience sample of shoppers from Save Mart Supermarket stores in both cities. Participants from Turlock were younger, had individuals who reported as non-Portuguese, had lower rates of college-level education and reported a lower median household income (USD 56,639), compared with participants from Livermore, who were mostly over 65 years of age with more college-educated individuals and a higher median household income (USD 116,942) [112]. Participants from Turlock had a higher adherence to the Mediterranean diet compared with participants from Livermore, probably due to their familiarity with the food components of the Mediterranean diet, which is similar to that found in Portuguese food [112]. However, convenience, sensory appeal and health were significant barriers for the Turlock participants in adhering to the Mediterranean diet [112]. The cultural food patterns of the Portuguese include fish, fruit, and cheese, wine and low amounts of processed foods. Couto et al. [112] attribute the high adherence in the Turlock participants to the “Healthy Immigrant Effect” from observations that immigrants are, on average, healthier than native-born participants. The association of high adherence to the Mediterranean diet with lower education are contrary to the results of other studies conducted in other parts of the US [109,112].

Adherence to the Mediterranean diet has been studied in Midwestern states within the US: among firefighters in in 44 fire stations in Indianapolis, Indiana [113,114] and in 11 fire departments in two Midwestern states where a young cohort of career firefighters participated in a cross-sectional study to determine the association of the Mediterranean diet with the risk of developing cardiovascular disease [115]. Mediterranean diet intervention in a cluster-randomized control trial was associated with improved lipid profiles and inflammation markers at one year and at six months following the intervention [114]. Similar data were obtained in a Mediterranean diet intervention study in a sub-cohort of firefighters in the “Feeding America’s Bravest” cluster-randomized controlled trial [113]. Food consumption frequency reporting by participants focused on the consumption of olive oil, vegetables, fruits, red meat/processed meats, butter/margarine, soda drinks, wine, legumes, fish/sea food, nuts, commercial sweets and sofrito in the 2020 study [114]. Fast food, sweet deserts, fried foods, ocean fish, breads and starches, non-alcoholic beverages and the type and frequency of alcoholic beverages were examples of foods reported in the questionnaires [114]. Measuring plasma levels of omega-3, which improved with Mediterranean diet intervention, supported compliance and adherence to the Mediterranean diet. Olive oil was associated with reduced inflammatory markers such as TNF alpha [113]. Barriers to adherence to the Mediterranean diet were not assessed in these studies.

Perceptions of the Mediterranean diet were evaluated by Angastinioti et al. [116] in a cohort of young adult university students, 18–25 years of age, from Cyprus and the US. Using focus groups and a Mediterranean diet score questionnaire, participants recorded their responses regarding foods consumed. Mediterranean diet adherence derived from participants’ self-reports determined that both groups had low to moderate adherence [116]. Although recognition of foods consumed in the Mediterranean diet was similar among the young adults from Cyprus and the US, the participants from Cyprus were familiar with more foods due to similarities in the Cypriot diet with the Mediterranean diet. However, there were inconsistencies in the knowledge of the health benefits of the diet [116]. Cognitive and motivational barriers such as the lack of food preparation skills and the need to have quick food were reported by US participants, who reported convenience as a major factor impacting food choices. Participants from both countries identified sensory and financially related factors such as taste and high food cost as influencing their food choices [15,116]. Food taste had a stronger impact on food choices than the health benefits and nutritional value of the foods [116].

Hardin-Fanning [117] identified the contribution of poor dietary habits to high rates of heart disease. Breathitt County in southeastern Kentucky, a rural Appalachian community, is a food desert where 54% of residents have difficulty accessing healthy foods. The PRECEDE–PROCEED model was used to evaluate and identify barriers to dietary changes and to determine factors that would facilitate community engagement with the introduction of the Mediterranean diet. Among the top three barriers to adopting and adhering to the Mediterranean diet in the Appalachian community were personal habits, limited access to healthy foods and the cost of the foods [117]. These barriers fall into the categories of Availability/Accessibility and Financial identified by Tsofliou et al. [15]. Motivational and sociocultural factors influenced additional barriers such as the difficulty of preparing foods, limited knowledge of the health benefits of foods, family members’ attitudes toward food and difficulty in incorporating healthy foods into meals [15,117].

## 9. Adherence to the Mediterranean Diet in US Racial and Minority Populations

Very few studies have been performed to understand the association of the Mediterranean diet with the risk of stroke, cardiovascular disease, obesity, and metabolic syndrome among racial and minority populations in the US. The few studies that have been conducted include population studies and clinical trials. Among clinical trials with Mediterranean diet intervention, the number of studies is very small compared with the studies carried out with predominantly white participants in the US. Sotos-Prieto and Mattei [13] have reviewed some of these studies, and Knight et al. [16] studied the Mediterranean diet in the Stroke Belt of the US. In the studies described below, ethnic and minority participants were enrolled in studies where the effects of adherence to the Mediterranean diet were evaluated. Barriers to adherence were not always identified. However, in the studies reviewed by Sotos-Prieto and Mattei [13], adaptations to the Mediterranean diet food components were made in the observational and intervention studies due to participant unfamiliarity with the foods. These studies are examined below in the context of the types of foods reported by study participants and whether any barriers to consuming specific food components identified as part of the traditional Mediterranean diet were reported or whether there were barriers to accepting the Mediterranean diet itself.

A number of studies were performed by Gardener et al. [118,119,120,121] examining adherence to the Mediterranean diet and cardiovascular disease in a prospective cohort study of multiethnic participants in Northern Manhattan, New York City. The Northern Manhattan Study (NOMAS) investigated risk factors and prognosis for stroke and adherence to the Mediterranean diet. The 3298 participants included Hispanics (63%), non-Hispanic Blacks (20%) and non-Hispanic whites (15%) [118]. Assessments of food intake were based on the MeDi score. Participants with high adherence to the Mediterranean diet had a lower risk of ischemic stroke, myocardial infarction and vascular death along with a lower left ventricular mass compared with the group with low adherence after a follow up of nine years [118]. The dietary pattern in NOMAS did not include typical food components found in the traditional Mediterranean diet. However, legumes, along with fish and alcohol, were part of the diet leading to a lower risk of developing vascular events, suggesting that the Mediterranean diet food components provided a protective effect. Legumes and pulses, along with red wine, olives and olive oil, are important components of the Mediterranean diet [30]. However, the types of fish, legumes and alcohol were not described in the study [118]. Differential effects associated with race or ethnicity were not observed [118]. In the evaluation of the Mediterranean diet and of the benefits to white-matter hyperintensity (WMH) in the same NOMAS cohort, modifications were made for the inclusion of Hispanic dietary food items [119]. High Mediterranean diet adherence was associated with a lower burden of WMH, even though the foods reported did not reflect those found in the traditional Mediterranean diet [119]. Reductions in atherosclerosis progression [120], a decrease in the risk of increased left ventricular mass [121] and protection against intracranial large-artery stenosis (ICAS) [122] in the NOMAS cohort were associated with adherence to the Mediterranean diet. Although adherence to the Mediterranean diet was higher among Hispanics in the cohort, barriers to adherence among the multiethnic group of participants was not explored [118,119,120,121,122].

In the Multi-Ethnic Study of Atherosclerosis (MESA), participants enrolled included white, African American, Hispanic and Chinese US participants. The Food Frequency Questionnaire (FFQ) was used to assess adherence and nutrient uptake from food components in the Mediterranean diet. Participants included men and women aged 45–84 years [123,124]. Participants in the MESA, a prospective cohort study, were recruited from Baltimore, Maryland; Chicago, Illinois; Forsyth, North Carolina; Los Angeles, California; Northern Manhattan and Bronx, New York; and St. Paul, Minnesota [124]. High adherence to the Mediterranean diet was associated with improved glucose levels and reduced plaque. Abiemo et al. [123] identified vegetables, legumes, whole grains, nuts and fruits. In addition, red and processed meats, monounsaturated and saturated fatty acid intake, dairy products, fish and alcohol were reported by participants [123]. Whether the food components were the same as what would be expected in a traditional Mediterranean diet is unclear. Legumes, nuts and monounsaturated fatty acids are components of the Mediterranean diet [2,30]. Vegetable consumption was the only Mediterranean diet component related to reduced plaque thickness, and older age was related to shorter telomere length in African Americans and Hispanics regardless of diet and other lifestyles [123]. In the same cohort, improvements in the left ventricular (LV) volume, ejection fraction and stroke volume were observed in participants with high adherence to the Mediterranean diet [124]. Barriers to adherence to the Mediterranean diet were not explored in both investigations.

The modified Mediterranean diet score was used with African American and white participants aged 18–30 years of age in the Coronary Artery Risk Development in Young Adults (CARDIA) study. High adherence to the Mediterranean diet resulted in a lower incidence of metabolic syndrome and associated health conditions compared with participants with low adherence [125]. A combination of foods included in the Mediterranean diet and foods added to increase adherence were reported in the questionnaire, suggesting that there were unacceptable or unfamiliar foods that might have been rejected by participants. Study participants reported legumes, vegetables, whole grains, and fruits. Both polyunsaturated, monounsaturated and saturated fatty acids were reported along with nuts, fish, eggs, poultry and alcohol. These were considered to be Mediterranean diet foods [125]. The source of the unsaturated fatty acids was not explained, and the types of vegetables were not provided [125]. Steffen et al. [125] recognized foods such as red and processed meats, along with fried foods, sweet juices, sweet beverages, diet beverages, coffee, refined grains, sauces and snack foods among the food items consumed by participants, which were not part of the Mediterranean diet. Participants were used to the food items in their diets and included them in their responses. No differential results were observed among racial or ethnic participants in the timeframe of the study, during which responses to the dietary history questionnaire were collected at baseline, 7 and 20 years [125].

The Building Research In Diet and Cognition study enrolled a predominantly African American cohort (91.4%) in a randomized clinical trial in Chicago, IL, with Mediterranean diet interventions in two of the three arms [126]. The study characterized the baseline characteristics of obese older African Americans and the effects of adherence to the Mediterranean diet on cognition in such areas as higher attention and information processing (AIP) and higher executive functioning (EF) [126]. Adherence to the Mediterranean diet was self-reported using a dietary survey instrument and showed that adherence to the Mediterranean-diet-like pattern was associated with higher AIP and EF. The specific foods of the Mediterranean diet and any cultural adaptations to the diet were not specified since food components of the diet were not reported [126].

The Washington Heights–Inwood Community Aging Project (WHICAP) study was a cross-sectional analysis that evaluated the association of the Mediterranean diet with leukocyte telomere length among white, Hispanic and African American participants [127]. Adherence to the Mediterranean diet was associated with leukocyte telomere length in whites but not in African American and Hispanic participants. Guo et al. [128] reported that non-Hispanic whites had a greater alcohol and fruit intake compared with non-Hispanic blacks, who reported more meat consumption, and Hispanics, who reported the consumption of more cereals, legumes and dairy products [128]. Consuming a wider variety of food types may be associated with a higher Mediterranean diet adherence compared with low variety and low adherence, even when the foods consumed are not typical Mediterranean food components but have a similar nutritional value [128]. Participants of WHICAP were evaluated for the association of Mediterranean diet adherence with risks of activities of daily living (ADL) disability. Determination was made by sex and by race and ethnicity [128]. Evaluations for single foods in the Mediterranean diet have resulted in inconsistent data, with some studies showing dairy products not significantly associated with the risk of instrumental activities of daily living (IADL) disability or basic activities of daily living (BADL) disability. Consumption of low amounts of dairy products was associated with higher risks of IADL disability but not for overall BADL and IADL (B-IADL) disabilities [128]. Factors influencing the selective consumption of specific food items and any barriers impacting selection were not addressed in the study.

A twelve-week Mediterranean diet intervention study (The Healthy Hearts Program) was performed to evaluate the association of the Mediterranean diet with the risk for cardiovascular disease [129]. The study was performed in a region of the southeastern US at high risk of obesity, cardiovascular disease and physical inactivity and low adherence to the Mediterranean diet. Thirty participants were stratified into two arms consisting of the American Heart Association and the Mediterranean diet arms. The Mediterranean diet arm was supplemented with extra-virgin olive oil (EVOO) and mixed nuts as well as nutrition education. Willis [129] acknowledged the challenge posed by Mediterranean diet food components such as EVOO and nuts, which have been identified as food barriers to Mediterranean diet adherence in the Stroke Belt of the US [129]. Nutrition education was associated with high adherence to the Mediterranean diet, and improvements in lipid and blood glucose profiles and a decrease in cardiovascular risk factors were observed [129].

Adherence to the Mediterranean diet its association with the risks of adverse pregnancy outcomes, including pre-term births and low birth weight, was evaluated in a prospective multicenter cohort study [130] and in a secondary analysis of the Boston Birth Cohort (BBC) [131]. Unfavorable birth outcomes were associated with low adherence to the Mediterranean diet in the BBC. Participants in the BBC were low-income and predominantly African American [131]. High adherence to the Mediterranean diet at the time of conception was positively associated with lower odds of adverse pregnancy outcomes in the multicenter study, where an association was seen in the types of foods consumed [130]. Low consumption of red and processed meat and increased consumption of vegetables, legumes, fruits, whole grains and fish were associated with positive pregnancy outcomes [130]. The multicenter study consisted of 10.5% non-Hispanic black, 16.6% Hispanic, 4.3% Asian and 63.9% non-Hispanic white participants. Adherence to the Mediterranean diet was beneficial for participants as no significant differences among race and ethnicity were seen in pregnancy outcomes [130]. However, barriers to Mediterranean diet adherence were not investigated.

## 10. Barriers to Adherence to the Mediterranean Diet in Racial and Ethnic Minority Populations in the US

In the studies summarized below, participants provided self-reported food intake information using survey instruments and food questionnaires, and those enrolled in randomized clinical trials consumed foods that were adapted to the Mediterranean diet and not a part of the traditional Mediterranean diet. Yet, in order to study perceived barriers and facilitators, in some cases, only one or two food items were included in the studies. Barriers to the adoption of and adherence to the Mediterranean diet were identified in the studies discussed below and summarized in Table 3.

### African Americans

Adherence to the Mediterranean diet in the US is low compared with the adherence in countries in the Mediterranean region, particularly in the Stroke Belt of the US [16]. Unfamiliarity with the lifestyle and food patterns of the Mediterranean, differences in food preparation and tastes, and a lack of understanding of the health benefits of the foods in the Mediterranean diet present major barriers toward acceptance of and adherence to the diet [16]. Study conclusions indicating high acceptance of the Mediterranean diet in the US base their conclusions on studies involving participants with high educational levels, high income and residence in predominantly white populations of western and northeastern coastal regions of the US [78]. Chen et al. [136] employed geographic information system (GIS) techniques to investigate Mediterranean diet adherence in the US in order to determine factors predicting adherence. Using data from the Reasons for Geographic And Racial Differences in Stroke (REGARDS) study [138], consisting of a cohort of non-Hispanic black and white participants aged more than 45 years of age, they identified geographic differences for Mediterranean diet adherence across 48 states in the US and Washington DC. Adoption of and adherence to the Mediterranean diet varies across the US and follows a geospatial and geographical distribution, where the highest levels of adherence are reported in the western coastal areas of California, southeastern Tennessee, northern Georgia, southern Florida, southeastern Pennsylvania, New Jersey, New York City, Connecticut and Massachusetts [136]. The areas of lowest Mediterranean diet adherence were in the Stroke Belt in the southeastern US and included Arkansas, Louisiana, northern Mississippi, north central Alabama, western Tennessee, southwestern Georgia and eastern North Carolina. Within the low-adherent populations in the southeast region, participants from urban communities with low poverty, high median household incomes and lower percentages of non-Hispanic white and black participants were more adherent. Poverty, low household median incomes and a higher percentage of non-Hispanic black participants resided in low-Mediterranean-diet-adherent communities [136]. Low Mediterranean diet adherence was also reported in eastern, northern and central regions that included southern Michigan and northern Indiana [78,136]. Convenience, sensory factors and health were reported as barriers to the Mediterranean diet in the Stroke Belt compared with other regions [78]. Eating the Mediterranean diet is associated with a lower risk of developing cardiovascular disease, obesity, metabolic syndrome, type 2 diabetes, cancers and neurodegenerative diseases, including Alzheimer’s and Parkinson’s disease. Investigations of adherence in other regions of the US have focused on food intake reports by participants enrolled in studies investigating adherence in association with risks for cardiovascular disease and improvements in markers of inflammation. The specific types of foods consumed are reported in general categories without specific identification of the foods consumed, posing a major challenge when studies are compared. Differences in food scoring instruments also gave inconsistent results [13]. Studies investigating barriers to the adoption of and adherence to the Mediterranean diet in the US are few. Palatability of the foods in the Mediterranean diet, lack of knowledge for food preparation and the predominance of olive oil as a major source of fats are major barriers to acceptance and adherence [16]. The unavailability of specific food items in grocery stores and supermarkets, the lack of access to fresh fruits and vegetables and the high cost of Mediterranean diet food components are also barriers to adopting and adhering to the Mediterranean diet [136,137]. Participants enrolled in most US studies do not generally include racial and ethnic minority populations, and the food intake questionnaires may not reflect the foods eaten by this population [13].

Bottcher et al. [135] assessed adherence to the Mediterranean diet by validating a Mediterranean Diet Nutrition Knowledge (MDNK) survey using a new screening tool developed for use in the Stroke Belt in the cities of Auburn and Opelika in the state of Alabama in the southeastern US. A validated short Mediterranean Diet Adherence Screener (MEDAS) was also used in the survey. Participants were shoppers at supermarkets and at farmers’ markets and included university students and older adults over the age of 65 years with higher levels of education. Young adults aged 18–24 years shopped more at supermarkets compared with the older adults, who shopped at the farmers’ markets [135]. The young adults had a lower adherence to the Mediterranean diet, with lower MEDAS scores reflecting a lower consumption of olive oil, fruits and vegetables, protein and dairy compared with the older adults who shopped at the farmers’ market and who showed higher adherence [135]. Mediterranean diet adherence was low (40%) to medium (48.7%) among the total participants, and there was no significant difference among the participants from the two cities. The study authors identified obesity and access to grocery stores as barriers to adherence to the Mediterranean diet in the Stroke Belt [135]. Previous exposure to formal nutrition knowledge and shopping at farmers’ markets were associated with high adherence to the Mediterranean diet in both the MEDAS and MDNK scoring system [135]. Knight et al. [16] conducted a study that evaluated perceived barriers and benefits to adopting and adhering to the Mediterranean diet. Participants were grouped into the Stroke Belt group (*n* = 305), California (*n* = 489) and other US groups (*n* = 435). The California group served as a reference group due to its greater adherence to the Mediterranean diet and similarities in the climate to the Mediterranean region [16,136]. The Stroke Belt region consists of eleven southeastern states in the US identified as an area of low Mediterranean-diet adherence using geospatial analysis of the US [136]. In the study by Knight et al. [16], knowledge, convenience, sensory appeal and health were major barriers to the adoption of and adherence to the Mediterranean diet in the Stroke Belt group, despite recognizing the potential benefits of weight loss through adherence to the Mediterranean diet. Sensory appeal of the Mediterranean diet food components was a major perceived barrier preventing acceptance of the diet in the Stroke Belt region [16]. Compared with the California group and other US groups, the Stroke Belt group perceived the Mediterranean diet to be unhealthy since they had the most decreased score in the perceived health barrier [16]. Survey instruments used for scoring adherence to the Mediterranean diet in the US have been designed to capture responses that reflect the food intake of the primarily white participants and do not reflect the food patterns of racial and ethnic minority populations [16].

Participants in the Reasons for Geographic and Racial Differences in Stroke (REGARDS) study were from 48 states and Washington DC in the US. The longitudinal study cohort consisted of 30,239 black and white men and women [138,139]. The study evaluated adherence to the Mediterranean diet and the risk of sudden cardiac death based on foods similar to those that are found in the Mediterranean diet, foods that represent the standard American diet and foods associated with Southern cuisine, such as fried foods, egg dishes and sweetened beverages [139]. High adherence to the Mediterranean diet was associated with a lower risk of sudden cardiac death compared with low adherence in all racial groups. Food components reported include vegetables, fruits, lean meats, fish, nuts, yogurt, organ meats, Chinese food, Mexican food, red meat, processed meats, sodium, dairy foods, grains and starches and alcohol [139]. Five dietary patterns identified in the REGARDS cohort—Southern, Plant-based, Convenience, Sweet/Fats, and Alcohol/Salads [140]—were represented in the food patterns observed in the study evaluating sudden cardiac death. A cross-sectional secondary analysis of the REGARDS cohort examined consumption of the Mediterranean diet and the association of obesity with the foods consumed. Access to healthy foods in grocery stores did not lead to greater adoption and adherence to the Mediterranean diet due a strong preference for processed foods, sugary beverages, beer and spirits over the fruits, vegetables, legumes, cereals, whole grains, rice, pasta, fish, low-fat dairy, poultry and water, which are more reflective of the food components found in the Mediterranean diet [136]. Using the REGARDS cohort, Gray et al. [132] investigated the association of dietary patterns with living in a food desert. Among the five dietary patterns identified in the REGARDS cohort (Southern, Plant-based, Convenience, Sweets/Fats and Alcohol/Salads), the Mediterranean diet was also examined using the Mediterranean diet score. Vegetables, fruits, beans and fish were components of the plant-based diet, compared with fried foods, added fats, eggs, egg dishes, sugar-sweetened beverages, organ meats and processed meats [132]. Living in a food desert was a barrier to Mediterranean diet adherence and was associated with low adherence to the Mediterranean diet and plant-based diets and high adherence to a Southern dietary pattern. Food deserts have populations with low income and low access to food, thereby creating barriers to a healthy diet such as the Mediterranean diet [117,132]. Subtle differences were observed among the populations studied regarding the effects of the plant-based and Mediterranean diets. Educational levels of participants did not influence dietary adherence in participants living in a food desert. African Americans were five times more likely to adhere to the Southern dietary pattern [132], which the authors attribute to cultural food choices.

Randomized clinical trials with dietary interventions have also examined factors that are barriers to the adoption of and adherence to the Mediterranean diet. Participants in the Heart Healthy Lenoir Project consisted of 65% African Americans from Lenoir County, North Carolina [141]. The study was designed to decrease health disparities in heart disease and stroke. Along with a Mediterranean diet intervention, weight loss through increased walking was evaluated. Dietary changes in the study included the consumption of healthy fats and carbohydrates. A modified Mediterranean diet, the Med–South, was employed due to food taste and food familiarity barriers. Among African American participants with and without diabetes, large improvements were observed in association with drinks, desserts and snacks consumption compared with whites [141,142]. African Americans had a slightly larger decrease in hemoglobin A1c levels than whites. Increased weight loss was seen in white participants with diabetes compared to African American participants with diabetes. Participants maintained their Med–South Diet scores at 24 months follow-up [141]. Food modifications in the study focused on incorporating the foods and tastes of regional cuisines that included Southern foods rather than participant food preferences based on race [142].

## 11. Hispanic Americans

The Boston Puerto Rican Health study, designed as a longitudinal study, examined the association of the Mediterranean diet with cardiovascular health [81]. A high adherence to the Mediterranean diet was associated with lower body mass indexes (BMIs), insulin levels, insulin resistance and C-reactive protein levels [81]. Other studies examined the adherence to the Mediterranean diet and cognitive performance in 1269 Puerto Rican adults 45–75 years of age from within the Boston Puerto Rican Health Study cohort [85]. High adherence associated with high scores was associated with better global cognitive function and a lower likelihood of cognitive impairment [85]. A study of the same cohort reported that high adherence to the Mediterranean diet was associated with lower depressive symptoms [83]. The same cohort was examined for effects of vitamin D on depressive symptoms along with adherence to the Mediterranean diet [133]. The studies indicated no association between vitamin D and depressive symptoms [133]. Various cultural, economic and social conditions affect the quality of food intake and dietary patterns that influence physical and mental health [83,133]. Adherence to the Mediterranean diet was evaluated using a Food Frequency Questionnaire (FFQ) designed and validated for Puerto Rican adults to reflect food differences in the traditional Puerto Rican diet. Adherence was associated with whole grains, vegetables, fruits, legumes, nuts, fish, the monounsaturated:saturated fat ratio, meat, poultry, dairy and alcohol [83]. Modifications made to the diet in the self-reported FFQ included the addition of foods such as plantain, avocado, mango, cassava, empanadas, and custard, which are part of the traditional Hispanic foods [83]. The specific types of foods consumed by participants, and whether they were traditional food components or alternate food components with similar nutritional value as those in the Mediterranean diet, were not indicated. However, the alternate foods incorporated suggest that food acceptability and unfamiliarity with the foods were barriers to adherence in the studies by Ye et al. [85] and Sahasrabudhe et al. [83]. In another Puerto Rican study by Mattei et al. [81], some specific traditional Puerto Rican foods were also substituted for foods normally found in the traditional Mediterranean diet while retaining some essential components of the Mediterranean diet. The foods included vegetables (mostly root crops and green bananas), beans and legumes, meats in homemade soup, orange juice, oatmeal, whole milk, corn oil and beer. The fish consumed included cod and canned tuna [81]. In this study, substitutions were made to the traditional Mediterranean diet food components due to barriers posed by participant unfamiliarity with the foods, leading to non-adherence to the Mediterranean diet. In a recent study, the Puerto Rican Optimized Mediterranean-like diet (PROMED) consisting of ethnically-tailored foods incorporated in a Mediterranean-like food pattern was shown to be an acceptable design for implementing nutrition intervention studies that may prove highly beneficial for the adherence to Mediterranean type diets by identifying foods that promote adherence [81]. Evidence-based diets like the Mediterranean diet are similar to other diets that share components of the Mediterranean diet with emphasis in increased consumption of vegetables, fruits, nuts and legumes with a reduction in sodium levels such as the Dietary Approaches to Stop Hypertension (DASH) or the Mediterranean-DASH Intervention for Neurodegenerative Delay (MIND) diet [143]. The inclusion of nutritionally equivalent foods from different ethnic groups might increase acceptability and help to promote increased adherence to the Mediterranean diet [81,143].

A version of the Heart Healthy Lenoir Project enrolled low-income Hispanic American young women in eastern North Carolina to evaluate the association of the Mediterranean diet with reduced cardiovascular disease risk. The study, known as the EnForma pilot study, evaluated cardiometabolic risk factors such as metabolic syndrome and obesity, which are high in Hispanic Americans [144]. The study focused on improving carbohydrate and lipid quality by using foods familiar to participants. Counseling on specific foods such as oils, dressings, nuts, fish, and meats was provided during the first visit by study participants. In subsequent visits, counseling on drinks, desserts, fruits and vegetables, grains and beans was provided. Study participants had a high adherence to the Mediterranean diet (100%) at the three-month follow-up. Nutrition counseling was effective in reducing barriers to adherence [144]. Similarly, the results of the EnForma–Diabetes feasibility study aimed at evaluating adherence to a Mediterranean dietary pattern among Hispanic American participants with type 2 diabetes reported acceptance of the diet [84]. Interviews assessed barriers that participants and Hispanic American food preparers might have regarding the nutritional intervention and food preparation, respectively. Information on oils, dressings, nuts, fish, and meats were provided during counseling sessions which helped to lessen barriers of food unfamiliarity for participants and food preparers [84].

The Viva Bien! Study is an intervention study that evaluated the adherence to the Mediterranean diet along with increased physical activity and management of stress among 280 Latinas with type 2 diabetes, who were stratified into two arms. The first arm consisted of “Usual care” only and the second arm consisted of “Usual care” plus “Viva Bien!” The barriers to the adoption of and adherence to the Mediterranean diet consisted of cultural food differences. Diverse Latin American foods were combined with food components of the Mediterranean diet, along with control of portion sizes. Mediterranean diet potluck dinners helped with the acceptance of the diet as participants became aware of Mediterranean diet food components. In addition to the dietary changes, supportive resources and smoking cessation were also incorporated into the study. Modest decreases in hemoglobin A1c levels were observed [145,146]. Other factors leading to improved adherence to the Mediterranean diet among the study participants include cooking demonstrations, education and family support.

## 12. Pacific Islander Americans and Asian Americans

There are very few studies evaluating Mediterranean diet adherence with various chronic non-communicable diseases among Pacific Islander Americans and Asian Americans. A multi-ethnic study (MEC) designed as a longitudinal study to understand the association of the Mediterranean diet with cancer and type 2 diabetes included men and women aged 45–75 years of age. Participants included whites, native Hawaiians and Japanese Americans. Higher adherence to the Mediterranean diet resulted in 13–28% lower risk of type 2 diabetes in white men but not in other ethnic groups or among white women [147]. A lower risk of type 2 diabetes was also identified in whites compared with African Americans, Japanese Americans, Latinos and Native Hawaiians in association with high adherence to an alternate Mediterranean diet (aMeD) [148]. Differences in glucose metabolism may contribute to the differences observed [148]. Jacobs et al. [149] showed that high adherence to the alternate Mediterranean diet was associated with lower mortality in colorectal cancer only in African American women, with nuts and legumes being important food components in the alternate Mediterranean diet. The foods consumed by participants included vegetables (without potatoes), whole grains, fruits, nuts, legumes, fish, red meat, processed meat and alcohol and had a high MUFA: SFA ratio. Cultural differences in the dietary habits and biological differences in the metabolism of participants may have played a role in the differences observed [148].

## 13. Native Americans and Alaskan Natives

Studies examining the impact of the Mediterranean diet on the health of Native American Indians and Alaska Natives are not available. The prevalence of rheumatoid arthritis (RA) and type 2 diabetes is high among several Native American and Alaskan Native populations [134,150,151]. Early-age onset, more severe symptoms and the pathogenesis of RA and its association with an increased frequency of the HLA DRB1*1402 allele suggests a genetic association for the high prevalence observed among American Indians and Alaskan Natives [150,151], particularly in women among the Yakima under the age of 35 years. However, environmental factors such as diet, tobacco, coffee, infections and sex hormones may also be contributing factors to the development of RA [150]. Due to the observation that the Mediterranean diet was associated with a low prevalence of RA in northeast Greece due to the contributions of omega-3 fatty acids in the diet, a similar association was sought in the Native American and Alaska Native populations. Among Alaska Natives, consumption of high levels of omega-3 fatty acids did not provide similar protection since the prevalence of RA remained high [150]. Stotz et al. [134] investigated theory-based determinants that would lead to the adoption of healthier food options aimed at managing type 2 diabetes. The options included the consumption of traditional foods that would provide nutritional benefits to reduce the risk of developing type 2 diabetes. The study uncovered barriers to healthy eating that are similar to those identified among other minority racial and ethnic groups [134]. In interviews and focus groups, the barriers identified include poverty, food insecurity, access to fast and processed foods, the lack of time to cook, the lack of cooking knowledge and skills, the high cost of healthy foods, stress, depression and other mental health, emotional and family challenges [134]. These barriers are compounded by the loss of traditional methods of farming, hunting, fishing and food preparation due to the loss of land ownership and environmental pollution in many of the communities [134]. Agricultural practices such as the cultivation of the *three sisters*, consisting of corn, beans and squash or pumpkin, are being revived to improve the nutrient quality of foods consumed and to revive sustainable ways to grow the crops [152]. Clayton and Ladi [153] discussed the Inuit diet, which originally consisted of the consumption of fresh or dried fish, whale meat and seal blubber as a major source of omega-3 fatty acids, polyphenols, phlorotannins and secoiridoids, which are major contributors to protection against inflammation, cancers, cardiovascular disease, neurodegenerative diseases and a host of other chronic diseases [153]. Although the Mediterranean diet was not discussed in both studies, adherence to traditional food items that are similar to foods in the Mediterranean diet or with similar nutritional value was recommended. The consumption of fish, game, walnuts, acorns and popcorn, along with beverages such as unsweetened tea and fruit infused water, provide similar nutritional benefits as the Mediterranean diet [134]. The availability of processed fish oils extracted and sold as capsules does not offer the same protection obtained from raw or dried fish, whale or seal blubber [134]. These observations call for more studies to be performed in partnership with communities within the Native American and Alaska Native populations to understand what traditional food items can provide similar nutritional benefits to those obtained from the Mediterranean diet that are required for reducing the risk of type 2 diabetes, RA, hypertension, renal failure and cerebrovascular disease, all of which are prevalent among Native American Indian and Alaska Native populations [134]. Furthermore, such research will determine how nutrition education can be tailored for each community to facilitate long-term adherence to healthy food preparation and consumption [151].

## 14. Perspectives

The studies examined in this review highlight the need to perform more studies that enroll participants of diverse racial and ethnic backgrounds and to develop dietary scoring instruments that reflect the foods consumed by different racial and ethnic populations in the US. Foods in the traditional Mediterranean diet were examined to identify those food components perceived to be strictly Mediterranean that are available in food markets and grocery stores. Food items such as cabbage, spinach, Swiss chard and potatoes are familiar produce items in most grocery stores. If individuals do not know what the traditional Mediterranean foods are and if nutrition intervention studies add only single components, such as olive oil, nuts or a single type of fruit or vegetable, the message conveyed is that the Mediterranean diet is based on single food items rather than on a synergy of food components, as would be found in other cultural dietary eating patterns. The view of the Mediterranean diet as a healthy diet has not been adequately conveyed to communities within the US. YouTube and Twitter (“X”) messages aimed at promoting the Mediterranean diet also add to the confusion with mixed messaging and inconsistent information [154,155]. The Mediterranean diet has been perceived as unhealthy [16] and the food components of the diet are regarded as unfamiliar and unacceptable to individuals in racial and ethnic minority populations that have different food cultures, cooking styles and that employ different food components in their meals. Controversies regarding the marginalization of non-Mediterranean cultural dietary patterns and cultural foods may be undermining the adoption of and adherence to the Mediterranean diet in the US [11,18]. A recent cross-national study using the 14-MEDAS scoring instrument evaluated Mediterranean diet adherence in five Mediterranean countries (Spain, Italy, Greece, Portugal and Cyprus) and two non-Mediterranean Balkan countries (Bulgaria and Republic of North Macedonia) [156]. A decline in adherence to the Mediterranean diet was observed, with dietary patterns becoming more westernized, with increased red meat consumption, the adoption of more sugary beverages and desserts and a decrease in the consumption of plant-based foods [156]. Incorporation of olive oil and more plant-based foods was highly reduced in the non-Mediterranean countries [156]. Efforts to educate populations in Mediterranean as well as in non-Mediterranean populations should evaluate the perceived barriers to and facilitators of adherence to the Mediterranean diet to assist with rational nutritional programs, policies and guidelines. The traditional Mediterranean diet should be conveyed as a non-restrictive eating pattern that incorporates a variety of plant-based foods, vegetables, fruits, olive oil, whole grains, legumes, nuts, poultry, fish and red meat in moderation. Eggs and dairy foods are consumed as part of the diet. The consumption of wine is not mandatory in the Mediterranean diet. Lack of nutrition knowledge, food access and food cost present major barriers to adherence to the Mediterranean diet as many of the communities with low adherence to the Mediterranean diet in the US are in food deserts and food swamps, where access to a variety of fresh fruits and vegetables is unavailable [157,158]. The hypothesis that food components of the Mediterranean diet are palatable, widely and easily accessible, acceptable, and affordable for individuals is not supported by the data reviewed in this paper. Food means different things to people, and just because a diet is deemed healthy and supported with scientific evidence does not guarantee that the diet will be embraced [159].

## 15. Conclusions

Before Ancel Keys, Giacomo Castelvetro encountered strong resistance in his efforts to change the eating habits of the English by advocating for the inclusion of vegetables (“mixed salads”) in the diet [159]. “The Sacred Law of Salads” proposed by Giacomo Castelvetro in the early 17th century, based on fresh vegetables and olive oil, has been demonstrated to be protective against HER-2-positive breast cancer [160]. In particular, the high antioxidant levels in the salads, such as from β-carotene, vitamins C and E, polyphenols, folate and monounsaturated fatty acids from the olive oil, are shown to repress HER-2 gene expression [160]. Some of the food components in the Mediterranean diet, such as garlic, fruits, vegetables and wheat bran, were frowned upon and thought to be dangerous and unhealthy [159]. The way in which food is marketed to create demand might be the most persuasive strategy to encourage the adoption of the Mediterranean way of eating while using alternative and similarly nutrient-dense foods in the diet. Detopoulou et al. [161] have developed an extensive food list to facilitate meal planning and food exchanges that will benefit individuals from different food cultures and backgrounds. The list incorporates food components in the traditional Mediterranean diet to help nutritionists and other health professionals counsel and educate clients [161]. Although developed for managing diabetes, the macromolecular content, nutrient density, glycemic load and fiber content of the foods have been evaluated and may prove very useful in promoting adherence to the Mediterranean diet [161]. Aliberti et al. [162] summarize characteristic dishes of the Blue Zone regions providing a resource that helps individuals distinguish the traditional Mediterranean foods from those of the Blue Zone regions. Limitations of this review include the omission of studies directly aimed at analyzing the nutritional content of the foods listed by the participants. In some of the studies, the mention of vegetables did not include specific types and there was no indication of whether the phytonutrient content is what should be expected for the Mediterranean diet. A search for the Mediterranean diet in association with African countries returned very few studies [90,91,92]. Due to the small number of studies and the differences in regional food culture, generalizations cannot be made for barriers and facilitators of adoption and adherence to the Mediterranean diet and is a strong indication for the need for more studies and inclusion of participants of diverse nationalities, racial and ethnic groups. The studies evaluated were identified in English-speaking databases. Studies in other languages reporting factors that serve as barriers to or facilitators of the adherence to the Mediterranean diet that would have aided our understanding of this important topic were not included. Finally, the studies reviewed enrolled adults of Mediterranean and non-Mediterranean communities that could independently provide responses to questionnaires and participate in educational programs that were part of the studies. Due to the scope of the review, many other important studies could not be included. The author apologizes for any omissions.

Recommendations for increasing the acceptance of and adherence to the Mediterranean diet include the following: (1) Provide nutrition education as a countermeasure to the barriers. The traditional Mediterranean diet should be explained, and a list of non-Mediterranean foods from other cultures with similar nutrition profiles and value should be included to increase variety and to increase the intake of fruits, vegetables, legumes, seeds and nuts. (2) Recipes and cooking instructions should be provided to help individuals feel confident in preparing and eating home cooked foods with family and friends. Cooking instructions will also help individuals overcome the unfamiliarity of using olive oil for cooking and in salads. (3) Part of the nutrition education and intervention during counseling should include a discussion on including herbs and spices in foods. This may require introducing individuals to herbs and spices that they have never used. (4) During counseling, it is important to know if individuals lack food access, cannot afford to buy healthy foods or if they live in a food swamp or food desert and if they participate in physical activity and have healthy sleep habits. (5) An understanding of the Mediterranean diet in the context of local communities should be viewed as a public health necessity, ensuring that the correct message is conveyed about the health benefits of incorporating Mediterranean dietary patterns into meals. (6) Efforts should be made to accommodate individuals with food allergies and food sensitivities. Due to the availability of variety in the Mediterranean diet and the availability of foods with similar nutritional value, nutrition education and intervention should include information about allergies and food insensitivities. The studies examined in this review show that high adherence to the Mediterranean diet improves health. However, this fact does not ensure that the diet will be readily accepted as indicated in the cited references in this review. Non-Mediterranean foods with equivalent health benefits should be recognized and included in nutritional counseling. The barriers preventing non-adherence to the Mediterranean diet identified among racial and ethnic minority participants in the US necessitate the need for more studies to be performed among racial and ethnic minority populations in the US.

## Figures and Tables

**Table 1:** Traditional Greek foods in recipes (1950s and 1960s).

Reference	Vegetables and Fruits	Herbs, Spices, Wine	Meat, Fish, Oils, Cheese
Wright [19]	Cabbage, spinach, Swiss chard, okra, eggplant, potato, lettuce, pea, green pepper, celery, eggplant, artichoke, beet, cucumber, carrot, scallion Pasta Lemon, olive Baked mixed vegetables (eggplant, tomato, zucchini, okra, parsley leaves) Eggplant moussaka Macaronia me Kima (Spaghetti with ground meat and tomato sauce)	Garlic, onion, leek, rosemary, dill, black pepper, allspice, nutmeg, oregano, thyme, bay leaf, marjoram, mint, cloves, cinnamon, black pepper, savory leaves, cumin, sesame seeds White wine, red wine vinegar	Lamb stew, pork and celery with avgolemono sauce (egg and lemon sauce) Marinated meatballs Beef Cuttlefish Extra virgin olive oil, feta cheese, milk, butter
Examples of Cretan dishes	Tourta (Meat pie), Horta (boiled greens with olive oil), Staka (homemade curd dip), Soupies me Patatas (cuttlefish, potato and olive stew)		

**Table 2:** Major foods of the Blue Zone regions and their health benefits. References: [40,42,43,44,45,46].

Blue Zone (BZ) Countries	Major Foods Characterizing BZs	Major Health Benefits from Foods
Ikaria, Greece BZ diet	Olive oil, wild vegetables, bitter greens, legumes, fruits, herbal teas, honey, Ikarian red wine, Greek coffee	Anti-inflammatory and antioxidative nutrients, phytonutrients, diuretics, micronutrients in wine and honey, high levels of polyphenols
Sardinia, Italy BZ diet	Olive oil, whole-grain sourdough bread, pasta, legumes, vegetables, red wine, meat, lard, cheese	Low-glycemic index foods, high levels of vitamin D and choline, antioxidative properties from foods and wine
Okinawa, Japan BZ diet	Canola oil, soybean oil, Okinawan sweet potatoes (purple sweet potatoes), seaweed, vegetables, bitter gourd, mulberry leaves, tofu, daikon, green papaya, water rich in calcium, small amounts of fish	Low-calorie, nutrient-dense with vitamins, minerals, phytonutrients
Nicoya, Costa Rica BZ diet	Corn, beans, squash (*the three sisters*), pumpkin, mango, papaya, magnesium and calcium in drinking water	Micronutrients, cholesterol control, antioxidants Niacin from corn Cholesterol control
Loma Linda, California USA BZ diet	Fruits, legumes, grains, vegetables (60% of diet), dried fruit, nuts, mostly vegan or vegetarian, very little fish, meat or chicken	Anti-inflammatory and antioxidative nutrients, phytonutrients
Crete, Greece Mediterranean diet	Olive oil, wild plants, bitter vegetables, purslane, walnuts, fruits, figs, fish, snails	Increased n-3 fatty acids, antioxidants and anti-inflammatory nutrients, phytonutrients, high levels of polyphenols

**Table 3:** Barriers to accepting and adhering to the Mediterranean diet among racial and ethnic minority populations in the US.

Barriers to Accepting and Adhering to the Mediterranean Diet	References
**Food barriers**	
Differences in food taste, sensory appeal, unpalatability of foods in Mediterranean diet	[16,78]
Unfamiliarity with Mediterranean food patterns	[16,81,84,85]
Cultural food choices, differences and food preparation differences	[16,117]
Predominance of olive oil and nut consumption	[16,129]
Differences in food choices	[132]
Unfamiliarity with Mediterranean foods	[133]
Lack of knowledge in food preparation and cooking skills, lack of time to cook	[16], [134] *
**Health barriers**	
Lack of understanding of health benefits of Mediterranean diet	[16]
Mediterranean diet considered unhealthy	[16]
Health factors	[78]
Obesity, lack of access to grocery stores	[135]
Mental, emotional and family challenges	[134] *
**Food preferences**	
Cultural food preferences	[16]
Fast and processed food preferences, sugary beverage preference	[134] *, [136]
**Food cost**	
Poverty, low household median income	[78,136]
High cost of healthy foods	[134] *, [136,137]
**Food access**	
Convenience	[78]
Food deserts	[117,136,137]
Food insecurity	[136,137]
Food security	[132,135]
Easy access to fast and prepared foods	[134] *

* Reference [134]: Barriers to the adoption of healthy food choices among Native Americans and Alaska Natives mirror the barriers reported for the adoption of and adherence to the Mediterranean diet. These have been included to highlight the need for studies to include all ethnic and minority populations in the US.

## Data Availability

The original contributions presented in the study are included in the article, further inquiries can be directed to the corresponding author.
